# Supplementary material for: Individual Health Management (IHM) for Stress—A Randomised Controlled Trial (TALENT II Study)
Source: Healthcare (Basel). 2025 Dec 4;13(23):3181. doi: 10.3390/healthcare13233181 (PMC12692462; doi:10.3390/healthcare13233181)
Supplement: Supplementary file 1 [file healthcare-13-03181-s001.zip › healthcare-3990669-supplementary.pdf]

Supplementary Materials

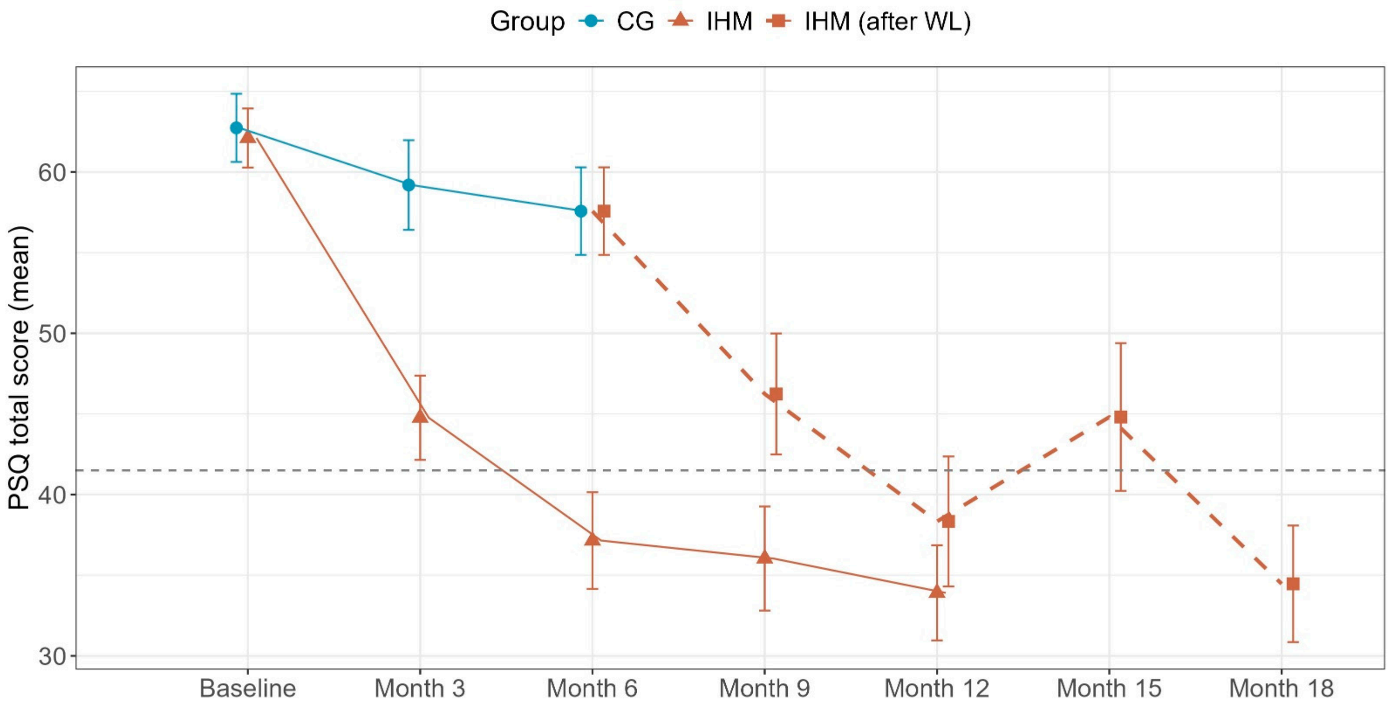

Figure S1. Cross-over analysis: A comparison of the mean differences for all time points (without using imputation). The dashed line shows the point at which things are no different. If the numbers are above the line, there has been a decline, and if the numbers are below the line, there has been an improvement. White circles show the mean, and black lines/circles show the median. The mean differences after M6 for the CG are the baseline values for the new IHM group.

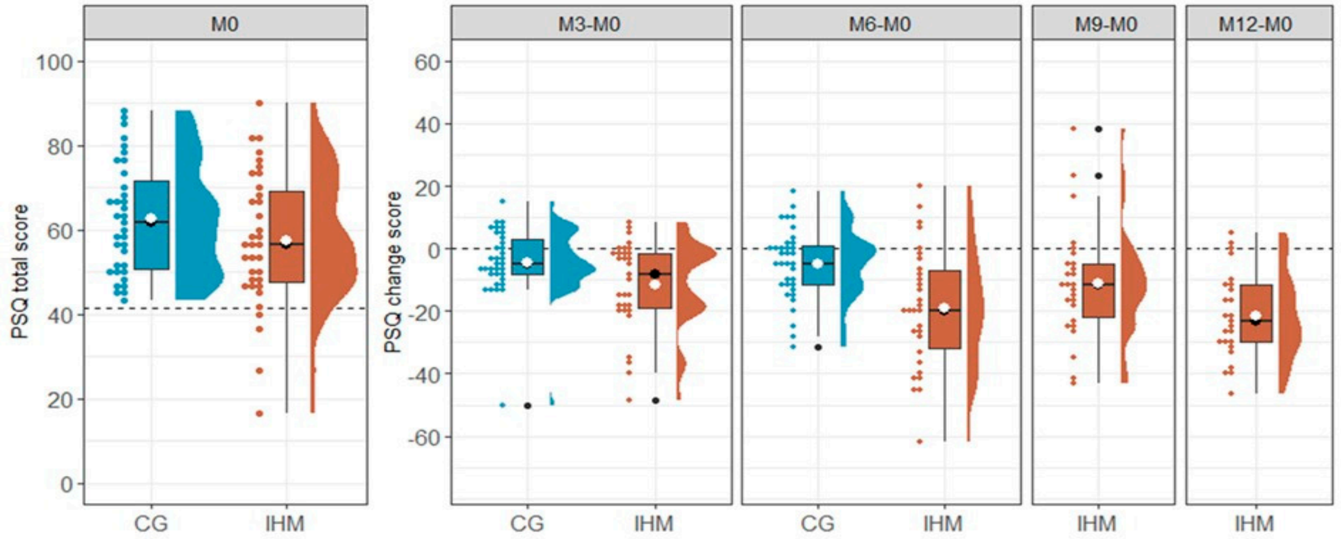

Figure S2. The mean total PSQ values at each time point (without imputation) The horizontal dashed line shows the threshold value below which the values are considered clinically normal. The solid lines represent the average values for the RCT groups (IHM and CG), while the dashed line shows the values for the CG after receiving the IHM following their time on the waiting list (WL).

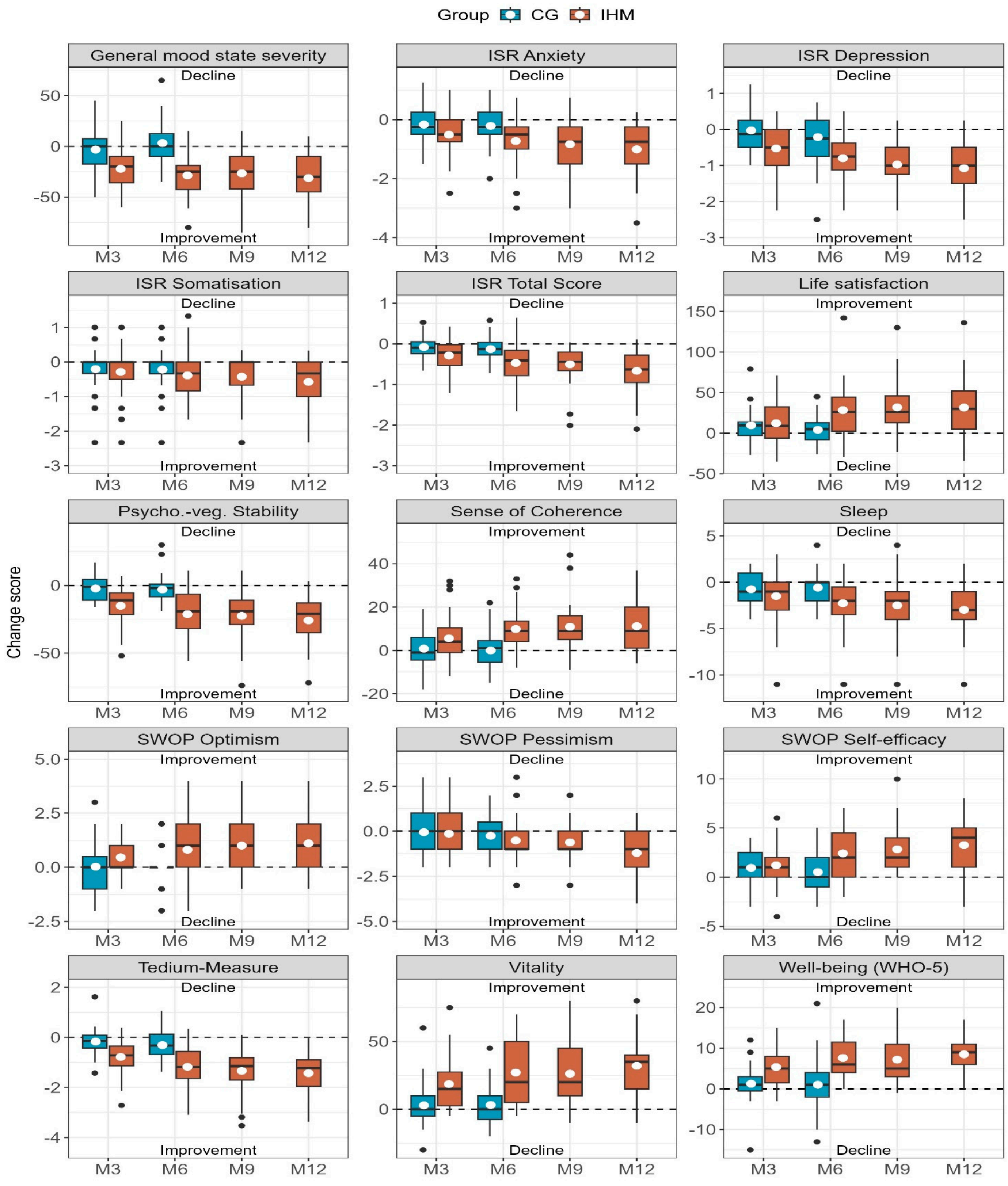

Figure S3. All outcomes: Distribution of mean differences (change score) at each time point per group (without imputation). The dashed line represents no change. White circles indicate the mean, black lines the median. M3, M6, M9 and M12 represent for the corresponding month of data collection

Table S1. Results for the stress profile.

	Intervention group (n = 38)				Control group (n = 39)				Between groups		ANCOVA
	Mean	SD	ΔM0	Cohen's d	Mean	SD	ΔM0	Cohen's d	Mean difference ΔM0 [95% CI]	Cohen's d	Parameter est. for group [95% CI]
Tedium-Measure (Burnout): total score											
M0	4.19	0.54			4.33	0.54					
M3	3.42	0.75	-0.77	-1.15***	4.19	0.71	-0.14	-0.25	-0.63 [-0.92; -0.35]	-1.03***	-0.69 [-0.98, -0.40]
M6	2.99	0.71	-1.20	-1.50***	4.06	0.70	-0.27	-0.46**	-0.94 [-1.26; -0.61]	-1.34***	-1.06 [-1.36, -0.75]
M9	2.75	0.68	-1.45	-1.71***							
M12	2.74	0.73	-1.45	-1.77***							
Tedium-Measure (Burnout): discouragement											
M0	3.81	0.74			4.08	0.62					
M3	3.09	0.95	-0.71	-0.96***	3.89	0.88	-0.19	-0.28	-0.52 [-0.87; 0.18]	-0.72**	-0.60 [-0.96, -0.23]
M6	2.61	0.85	-1.19	-1.41***	3.81	0.83	-0.27	-0.42*	-0.92 [-1.29; -0.55]	-1.22***	-1.08 [-1.45, -0.71]
M9	2.38	0.73	-1.42	-1.60***							
M12	2.46	0.82	-1.34	-1.53***							
Tedium-Measure (Burnout): exhaustion											
M0	4.92	0.72			4.91	0.68					
M3	3.96	0.95	-0.96	-1.04***	4.76	0.84	-0.16	-0.22	-0.81 [-1.20; -0.41]	-0.97***	-0.83 [-1.21, -0.45]
M6	3.60	0.93	-1.32	-1.20***	4.57	0.94	-0.35	-0.42*	-0.98 [-1.43; -0.51]	-0.99***	-1.02 [-1.45, -0.58]
M9	3.37	1.10	-1.55	-1.31***							
M12	3.28	1.06	-1.64	-1.35***							
Tedium-Measure (Burnout): loss of motivation											
M0	4.10	0.61			4.13	0.61					
M3	3.44	0.72	-0.66	-1.12***	4.12	0.71	-0.01	-0.01	-0.65 [-0.97; -0.34]	-1.05***	-0.68 [-1.00, -0.36]
M6	3.01	0.75	-1.09	-1.33***	3.97	0.72	-0.15	-0.24	-0.94 [-1.29; -0.58]	-1.26***	-1.00 [-1.33, -0.67]
M9	2.74	0.81	-1.36	-1.56***							
M12	2.66	0.68	-1.44	-2.08***							
Psycho-vegetative complaints total score											
M0	56.74	13.86			53.72	13.12					

M3	41.92	14.01	-14.82	-1.03***	51.98	14.86	-1.74	-0.19	-13.08 [-18.81; -7.35]	-1.08***	-13.03 [-18.43, -7.63]
M6	35.58	15.43	-21.15	-1.24***	50.77	15.08	-2.94	-0.25	-18.21 [-25.06; -11.36]	-1.24***	-17.99 [-24.34, -11.64]
M9	34.41	14.12	-22.33	-1.25***							
M12	31.07	14.45	-25.67	-1.48***							
	Psycho-vegetative complaint: sleep										
M0	6.45	2.52			6.23	2.47					
M3	5.05	2.22	-1.40	-0.54**	5.56	2.46	-0.67	-0.32	-0.73 [-1.89; 0.44]	-0.31	-0.71 [-1.76, 0.34]
M6	4.38	2.69	-2.07	-0.70***	5.79	2.36	-0.44	-0.22	-1.62 [-2.84; -0.40]	-0.64**	-1.66 [-2.79, -0.54]
M9	4.09	2.37	-2.36	-.73***							
M12	3.54	1.97	-2.90	-1.16***							
	Psycho-vegetative complaint: dizziness										
M0	3.61	1.94			2.90	1.80					
M3	2.62	1.82	-0.98	-0.49**	3.02	1.92	0.12	0.09	-1.11 [-1.94, -0.27]	-0.64*	-0.92 [-1.70, -0.14]
M6	2.13	1.72	-1.47	-0.64***	2.81	1.98	-0.09	-0.06	-1.38 [-2.29, -0.47]	-0.72**	-1.15 [-1.95, -0.34]
M9	2.04	1.48	-1.57	-0.77***							
M12	1.77	1.52	-1.83	-0.91***							

Note: This table summarises the results of independent samples t-tests comparing the intervention group (IHM) and the control group (CG), as well as the results of paired samples t-tests assessing changes within each group over time. For each time point, the following are reported: mean scores (± standard deviation, SD); mean change scores from baseline (ΔM0); effect sizes (Cohen's d: < 0.05 small effect, 0.5–0.8 medium effect, > 0.8 large effect); and p-values (significance: p < 0.05*, p < 0.01**, p < 0.001***). Between-group mean differences with 95% confidence intervals (CIs) are presented, along with the parameter estimate for the group factor from the ANCOVA model, adjusted for baseline scores and gender. All samples are based on the intent-to-treat (ITT) population. SD values and Cohen's d were calculated as the mean value across multiply imputed data.

Table S2. Results for the mental stress and burden (ISR)

	Intervention group (n = 38)				Control group (n = 39)				Between groups		ANCOVA
	**Mean**	**SD**	**ΔM0**	**Cohen's d**	**Mean**	**SD**	**ΔM0**	**Cohen's d**	**Mean difference ΔM0 [95% CI]**	**Cohen's d**	**Parameter est. for group [95% CI]**
	ISR: total score										
M0	1.13	0.51			1.07	0.42					
M3	0.87	0.47	-0.26	-0.66***	1.07	0.47	0.00	0.01	-0.27 [-0.44, -0.10]	-0.72**	-0.29 [-0.44, -0.14]
M6	0.72	0.43	-0.41	-0.85***	0.98	0.37	-0.09	-0.25	-0.32 [-0.52, -0.12]	-0.76**	-0.33 [-0.48, -0.17]
M9	0.66	0.41	-0.47	-0.99***							
M12	0.53	0.36	-0.61	-1.21***							
	ISR: depression										
M0	1.82	0.59			1.74	0.75					
M3	1.29	0.69	-0.53	-0.80***	1.75	0.88	0.01	0.01	-0.54 [-0.88, -0.21]	-0.78**	-0.55 [-0.88, -0.23]
M6	1.02	0.68	-0.80	-1.06***	1.53	0.72	-0.20	-0.26	-0.59 [-0.97, -0.22]	-0.76**	-0.59 [-0.92, -0.26]
M9	0.77	0.55	-1.06	-1.56***							
M12	0.71	0.58	-1.11	-1.62***							
	ISR: anxiety										
M0	1.49	1.00			1.26	0.87					
M3	0.97	0.85	-0.52	-0.78***	1.22	0.83	-0.03	-0.05	-0.48 [--0.82, -0.14]	-0.67**	-0.45 [-0.76, -0.15]
M6	0.73	0.71	-0.75	-0.82***	1.16	0.74	-0.10	-0.14	-0.66 [-1.05, -0.26]	-0.79**	-0.58 [-0.89, -0.28]
M9	0.67	0.70	-0.81	-0.89***							
M12	0.48	0.50	-1.00	-1.08***							

Note: The table summarises the results of independent samples t-tests comparing the intervention group (IHM) and the control group (CG), as well as the results of paired samples t-tests assessing changes within each group over time. For each time point, the following are reported: mean scores (± standard deviation, SD); mean change scores from baseline (ΔM0); effect sizes (Cohen's d: <.05 small effect, 0.5–0.8 medium effect, > 0.8 large effect); and p-values (significance: $p < 0.05$*, $p < 0.01$**, $p < 0.001$***). Between-group mean differences with 95% confidence intervals (CIs) are presented, along with the parameter estimate for the group factor from the ANCOVA model, adjusted for baseline scores and gender. All samples are based on the intent-to-treat (ITT) population. SD values and Cohen's d were calculated as the mean value across multiply imputed data

Table S3. Results for the psychological resource profile

	Intervention group (n = 38)				Control group (n = 39)				Between groups		ANCOVA
	**Mean**	**SD**	**ΔM0**	**Cohen's d**	**Mean**	**SD**	**ΔM0**	**Cohen's d**	**Mean difference ΔM0 [95% CI]**	**Cohen's d**	**Parameter est. for group [95% CI]**
	WHO well-being index										
M0	7.89	3.65			9.26	4.73					
M3	12.86	5.28	4.97	0.95***	9.96	5.34	0.71	0.16	4.26 [1.94, 6.58]	0.86***	3.85 [1.59, 6.11]
M6	15.03	4.28	7.14	1.42***	9.67	5.18	0.41	0.08	6.73 [4.31, 9.15]	1.28***	6.05 [3.88, 8.22]
M9	14.61	5.92	6.71	1.13***							
M12	15.82	4.25	7.92	1.65***							
	Vitality										
M0	35.00	15.55			37.69	15.34					
M3	52.08	20.54	17.08	0.82***	40.31	20.59	2.62	0.17	14.46 [5.34, 23.59]	0.79**	14.79 [5.96, 23.62]
M6	60.58	19.37	25.58	1.06***	40.17	19.06	2.47	0.15	23.10 [4.88, 13.35]	1.11***	22.49 [13.66, 31.32]
M9	60.05	22.37	25.05	1.03***							
M12	63.55	18.44	28.55	1.17***							
	Self-efficacy (SWOP)										
M0	12.21	2.73			11.41	2.45					
M3	13.26	2.70	1.05	0.47**	12.12	2.33	0.71	0.37	0.34 [-0.73, 1.41]	0.16	0.70 [-0.34, 1.75]
M6	14.59	2.53	2.38	0.89***	11.83	2.28	0.42	0.19	1.96 [0.74, 3.17]	0.78**	2.49 [1.41, 3.56]
M9	15.04	2.88	2.83	1.10***							
M12	15.47	2.44	3.26	1.23***							
	Optimism (SWOP)										
M0	5.32	1.36			4.74	1.25					
M3	5.84	1.28	0.52	0.50**	4.69	1.30	-0.05	-0.04	0.57 [0.03, 1.11]	0.51*	0.81 [0.28, 1.33]
M6	6.27	1.29	0.95	0.76***	4.76	1.29	0.01	0.01	0.94 [0.35, 1.53]0\	0.81**	1.18 [0.59, 1.77]
M9	6.54	1.15	1.23	0.93***							
M12	6.50	1.19	1.18	0.99***							
	Pessimism (SWOP)										
M0	5.00	1.19			5.00	1.19					
M3	4.83	1.26	-0.17	-0.13	4.89	1.15	-0.11	-0.09	-0.06 [-0.66, 0.54]	-0.05	-0.09 [-0.63, 0.44]
M6	4.42	1.53	-0.58	-0.36	4.75	1.18	-0.25	-0.22	-0.32 [-1.06, 0.42]	-0.23	-0.31 [-1.01, 0.39]

M9	4.36	1.56	-0.64	-0.43*							
M12	3.78	1.39	-1.22	-0.82***							
Sense of coherence: total score											
M0	54.53	9.16			54.54	9.44					
M3	60.10	11.74	5.57	0.51**	55.22	11.06	0.68	0.08	4.89 [0.25, 9.53]	0.50*	5.36 [0.85, 9.89]
M6	64.61	11.59	10.08	1.01***	54.76	11.18	0.22	0.02	9.87 [5.00, 14.73]	1.07***	10.04 [5.17, 14.91]
M9	65.90	10.61	11.38	1.03***							
M12	65.23	11.16	10.71	0.90***							
Sense of coherence: comprehensibility											
M0	20.11	4.90			20.21	4.37					
M3	22.15	5.20	2.04	0.40*	20.44	4.65	0.24	0.05	1.81 [-0.47, 4.09]	0.37	1.92 [-0.14, 3.99]
M6	24.28	5.68	4.17	0.97***	20.54	5.29	0.33	0.07	3.84 [1.25, 6.42]	0.83**	3.92 [1.37, 6.46]
M9	24.79	5.04	4.68	0.84***							
M12	24.25	5.25	4.15	0.76***							
Sense of coherence: manageability											
M0	16.26	3.59			16.23	4.56					
M3	18.40	4.44	2.13	0.50**	16.46	4.64	0.23	0.06	1.91 [-0.08, 3.90]	0.46.	2.09 [0.21, 3.96]
M6	19.14	4.44	2.88	0.61**	16.36	4.88	0.13	0.03	2.75 [0.53, 4.97]	0.64*	2.77 [0.60, 4.93]
M9	19.62	4.44	3.36	0.76***							
M12	19.85	4.71	3.58	0.74***							
Sense of coherence: meaningfulness											
M0	18.16	3.89			18.10	3.62					
M3	19.55	4.42	1.39	0.32.	18.32	3.83	0.22	0.07	1.17 [-0.64, 2.99]	0.31	1.33 [-0.36, 3.01]
M6	21.19	4.24	3.03	0.69***	17.85	3.55	-0.25	-0.09	3.28 [1.41, 5.16]	0.88***	3.37 [1.63, 5.12]
M9	21.49	3.83	3.34	0.76***							
M12	21.13	3.88	2.98	0.63***							
Life satisfaction: total score											
M0	33.34	27.37			29.97	26.70					
M3	42.51	25.83	9.17	0.33.	40.24	26.70	10.26	0.50**	-1.09 [13.32, 11.14]	-0.04	1.00 [-9.84, 11.84]
M6	59.90	28.28	26.56	0.75***	33.13	26.40	3.16	0.16	23.40 [9.63, 37.16]	0.81**	27.53 [15.77, 39.30]
M9	63.50	29.29	30.16	0.91***							
M12	62.93	32.75	29.59	0.83***							
Life satisfaction: friends/acquaintances											

M0	4.55	6.05			6.36	6.37					
M3	4.96	5.20	0.40	0.07	6.11	6.35	-0.25	-0.05	0.65 [-2.11, 3.42]	0.12	-0.26 [-2.78, 2.25]
M6	7.43	5.43	2.88	0.42*	4.62	4.78	-1.74	-0.30.	4.61 [1.56, 7.67]	0.73**	3.66 [1.31, 6.02]
M9	7.73	6.72	3.18	0.39*							
M12	7.92	5.44	3.37	0.49*							
Life satisfaction: leisure activities/hobbies											
M0	1.37	4.21			2.28	5.59					
M3	3.09	4.51	1.72	0.35.	3.72	5.02	1.44	0.28	0.28 [-2.20, 2.76]	0.06	-0.29 [-2.52, 1.93]
M6	6.06	6.16	4.69	0.73***	2.75	4.30	0.47	0.10	4.22 [1.48, 6.97]	0.74**	3.86 [1.43, 6.29]
M9	6.25	5.93	4.88	0.71***							
M12	5.69	5.98	4.32	0.66***							
Life satisfaction: health											
M0	-1.21	5.70			-0.74	6.24					
M3	3.88	5.93	5.09	0.71***	1.68	6.92	2.43	0.33.	2.66 [-0.91, 6.23]	0.37	2.15 [-1.07, 5.36]
M6	7.61	7.71	8.82	0.99***	2.12	6.21	2.86	0.46*	5.95 [2.00, 9.91]	0.78**	5.99 [2.35, 9.64]
M9	9.02	6.40	10.23	1.29***							
M12	8.88	7.07	10.09	1.12***							
Life satisfaction: income/ financial security											
M0	4.47	5.87			3.97	4.85					
M3	4.72	5.18	0.25	0.06	4.38	5.56	0.40	0.07	-0.15 [-2.75, 2.45]	-0.03	0.27 [-2.06, 2.60]
M6	7.09	5.33	2.61	0.34.	4.07	6.02	0.09	0.02	2.52 [-0.69, 5.73]	0.38	2.83 [-0.14, 5.81]
M9	7.04	4.95	2.57	0.42*							
M12	7.03	4.96	2.55	0.42*							
Life satisfaction: profession/work											
M0	2.21	6.16			1.74	5.86					
M3	2.78	5.71	0.57	0.13	2.69	5.73	0.95	0.17	-0.38 [-3.09, 2.34]	-0.07	-0.12 [-2.71, 2.47]
M6	4.54	5.27	2.33	0.37.	2.24	5.25	0.50	0.08	1.83 [-1.36, 5.01]	0.29	2.15 [-0.67, 4.96]
M9	4.32	7.61	2.11	0.28							
M12	5.23	5.48	3.02	0.47*							
Life satisfaction: housing situation											
M0	8.08	6.72			6.28	6.93					
M3	7.92	5.44	-0.16	-0.02	6.36	5.16	0.08	0.01	-0.24 [-3.48, 3.01]	-0.04	0.96 [-1.90, 3.81]
M6	8.54	6.10	0.47	0.06	5.26	6.54	-1.02	-0.16	1.48 [-1.86, 4.82]	0.22	2.82 [-0.16, 5.79]

M9	8.78	5.91	0.70	0.10							
M12	8.00	6.05	-0.08	-0.01							
Life satisfaction: family life/children											
M0	6.16	7.90			5.79	5.77					
M3	7.81	7.37	1.65	0.26	8.69	7.24	2.90	0.48*	-1.25 [-4.50, 2.00]	-0.20	-0.68 [-3.82, 2.46]
M6	8.89	7.41	2.74	0.28	7.03	7.35	1.23	0.21	1.51 [-2.57, 5.59]	0.19	2.37 [-1.33, 6.07]
M9	9.55	8.00	3.39	0.34.							
M12	10.39	6.97	4.23	0.47**							
Life satisfaction: partnership/relationship											
M0	7.71	8.62			4.28	7.23					
M3	7.36	7.98	-0.36	-0.04	6.60	7.71	2.32	0.34.	-2.68 [-6.54, 1.19]	-0.34	-1.01 [-4.52, 2.50]
M6	9.73	7.11	2.02	0.25	5.03	7.79	0.75	0.12	1.27 [-2.23, 4.77]	0.18	3.42 [0.26, 6.58]
M9	10.80	7.24	3.09	0.44.							
M12	9.80	7.82	2.09	0.22							

Note: The table summarises the results of independent samples t-tests comparing the intervention group (IHM) and the control group (CG), as well as the results of paired samples t-tests assessing changes within each group over time. For each time point, the following are reported: mean scores (± standard deviation, SD); mean change scores from baseline (ΔM0); effect sizes (Cohen's d: <0.05 small effect, 0.5–0.8 medium effect, >0.8 large effect); and p-values (significance: $p < 0.05$*, $p < 0.01$**, $p < 0.001$***). Between-group mean differences with 95% confidence intervals (CIs) are presented, along with the parameter estimate for the group factor from the ANCOVA model, adjusted for baseline scores and gender. All samples are based on the intent-to-treat (ITT) population. SD values and Cohen's d were calculated as the mean value across multiply imputed data.
